# Supplementary material for: Structural and evolutionary features of red algal UV sex chromosomes
Source: Genome Biol. 2025 Oct 7;26:341. doi: 10.1186/s13059-025-03797-y (PMC12502185; doi:10.1186/s13059-025-03797-y)
Supplement: Supplementary file 2 — Additional file 2: Fig S1-S6 Fig. S1. Codon usage bias (ENC') values for autosomal and sex-linked genes across four Gracilaria species. Fig. S2. Codon usage bias (ENC') values for autosomal and sex-linked genes across four Gracilaria species. Fig. S3. Expression of SDR genes. Fig. S4. Phylogenetic analysis suggesting the independent acquisition of certain gametolog pairs in G. vermiculophylla. Fig. S5. Wordcloud visualization of Gene Ontology (GO) terms enriched in sex-linked genes across all four Gracilaria species. Fig. S6. Evolutionary rates (measured as KN/KS) of male- and female-biased genes compared to unbiased genes across all four Gracilaria species (codeml, model M0, PAML4) showing no statistical difference between the groups (pairwise Wilcoxon test with Holm correction). [file 13059_2025_3797_MOESM2_ESM.pdf]

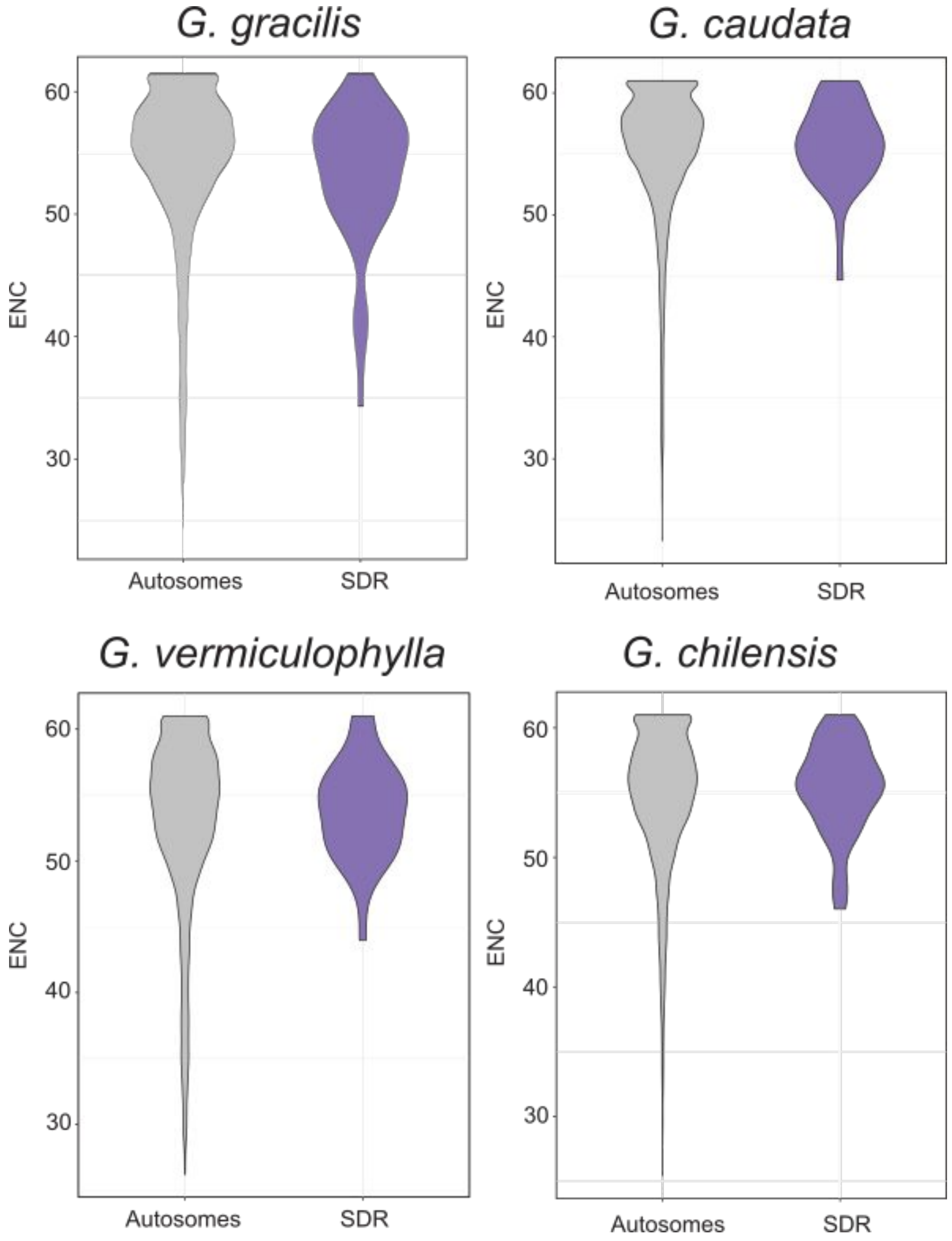

Fig. S1. Codon usage bias (ENC') values for autosomal and sex-linked genes across four *Gracilaria* species. The analysis revealed no significant differences between gene groups (Mann-Whitney U test, p-value > 0.05).

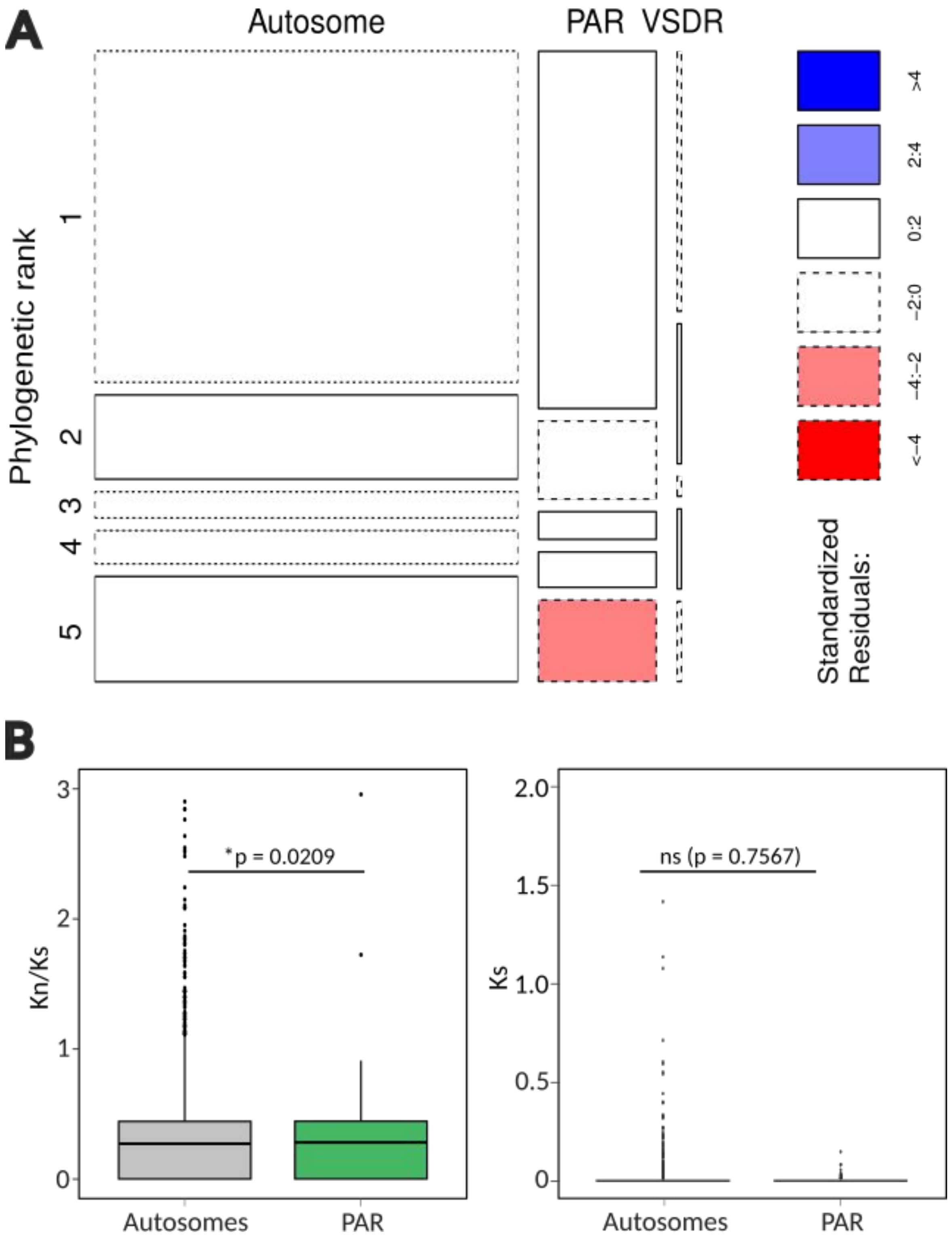

Fig. S2. Characteristics of the pseudoautosomal regions (PARs) compared to autosomes in *G. vermiculophylla*. A) Mosaic plot showing that the family-level (rank 5) genes are depleted in the sex chromosome. Phylogenetic ranks (1 - cellular organisms, 2 - Eukaryota, 3 - Rhodophyta, 4 - Rhodymeniophycidae, 5 - Gracilariaceae). B) Non-synonymous/synonymous (K_N/K_S and synonymous (K_S) substitution rates of the PAR and autosomal genes (permutation tests of the difference in the mean, 10k permutations).

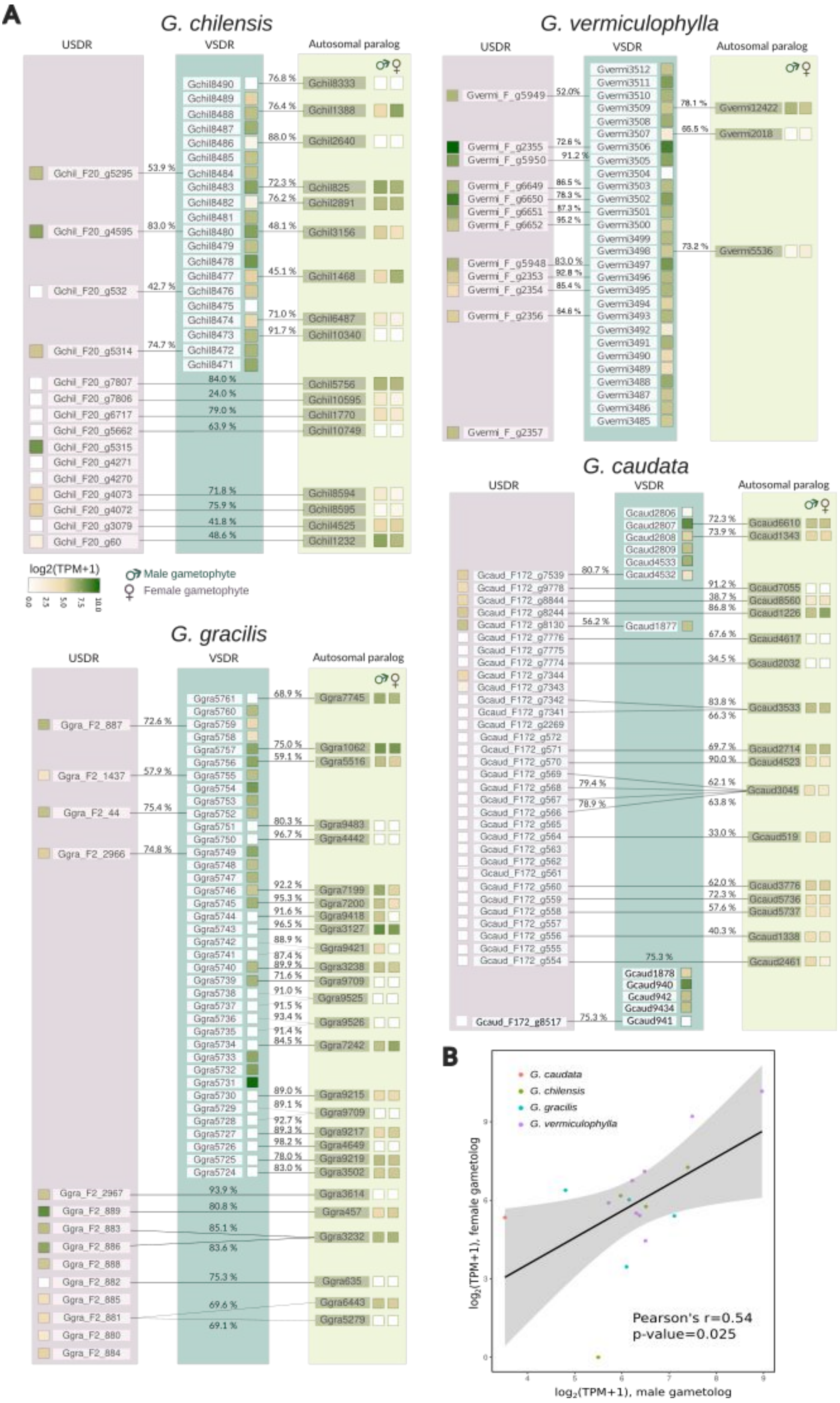

Fig. S3. Expression of SDR genes. A) Expression levels (log2(TPM+1)) of sex-linked genes (USDR in purple and VSDR in blue-green) and their autosomal paralogs (light green) in the four *Gracilaria* species. Percent of protein sequence identity are indicated above the lines. B) The correlation in transcript abundances between gametolog pairs (log2(TPM+1)) in males and females of the *Gracilaria* species.

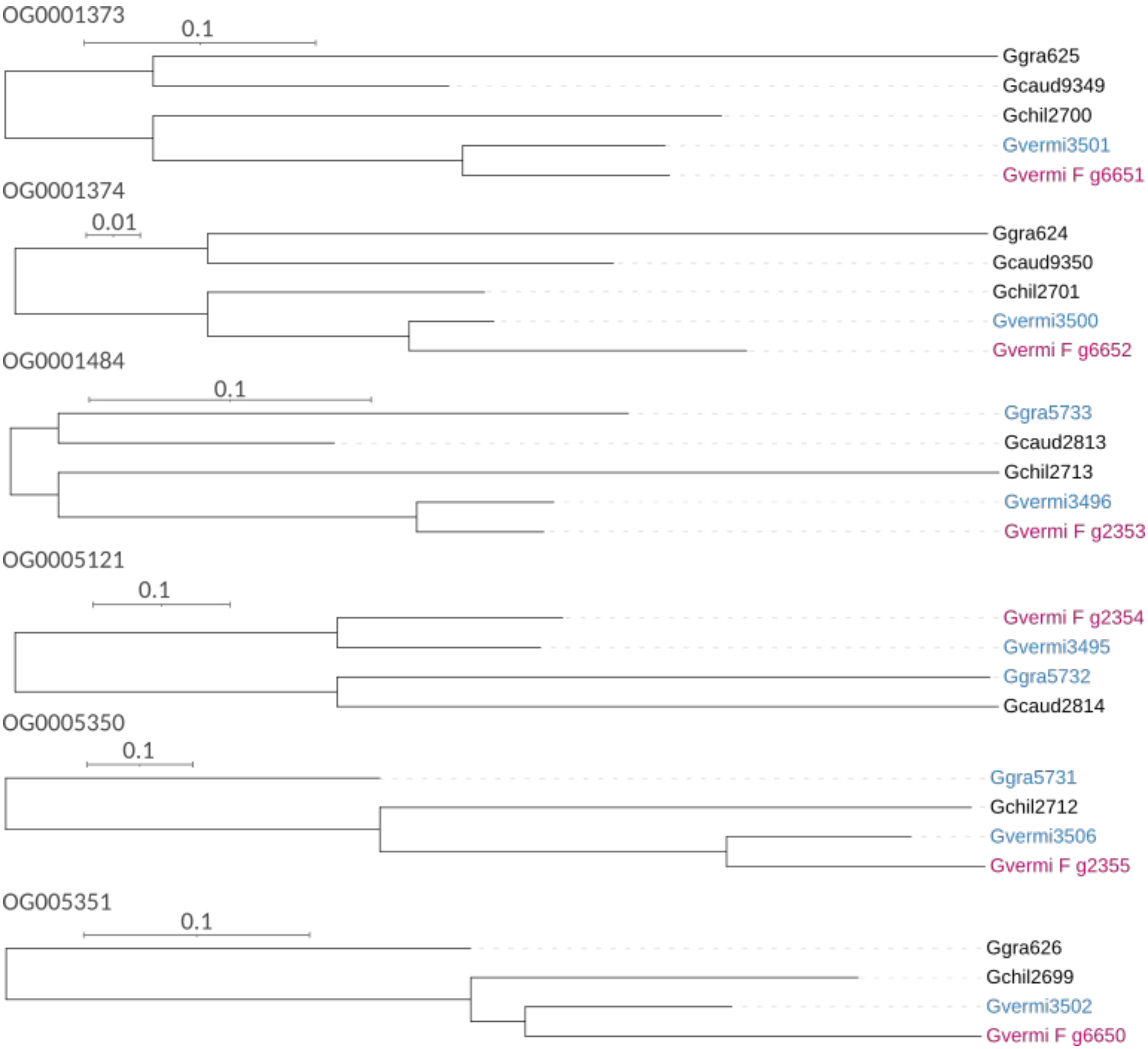

Fig. S4. Phylogenetic analysis suggesting the independent acquisition of certain gametolog pairs in *G. vermiculophylla*.

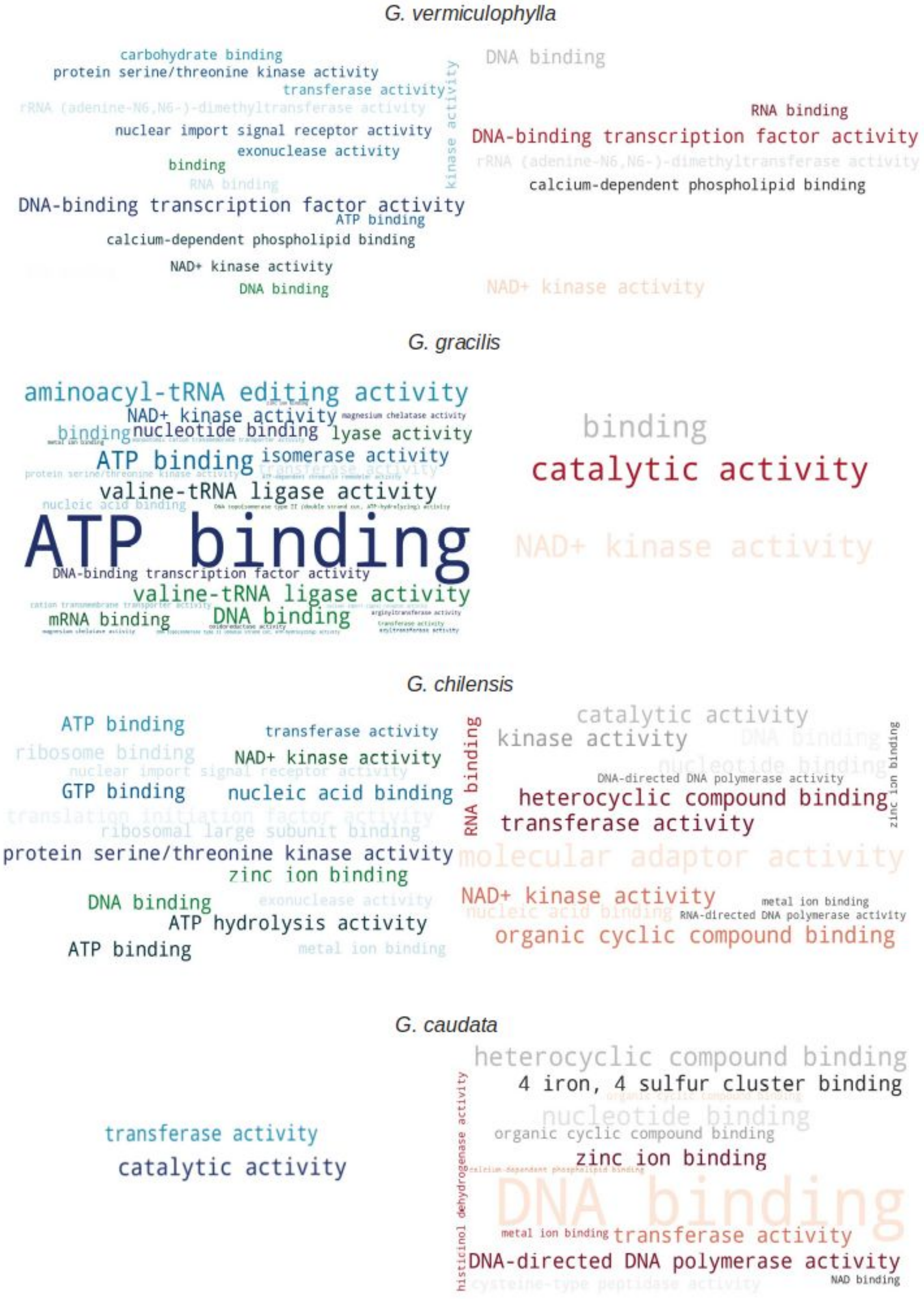

Fig. S5. Wordcloud visualization of Gene Ontology (GO) terms enriched in sex-linked genes across all four *Gracilaria* species.

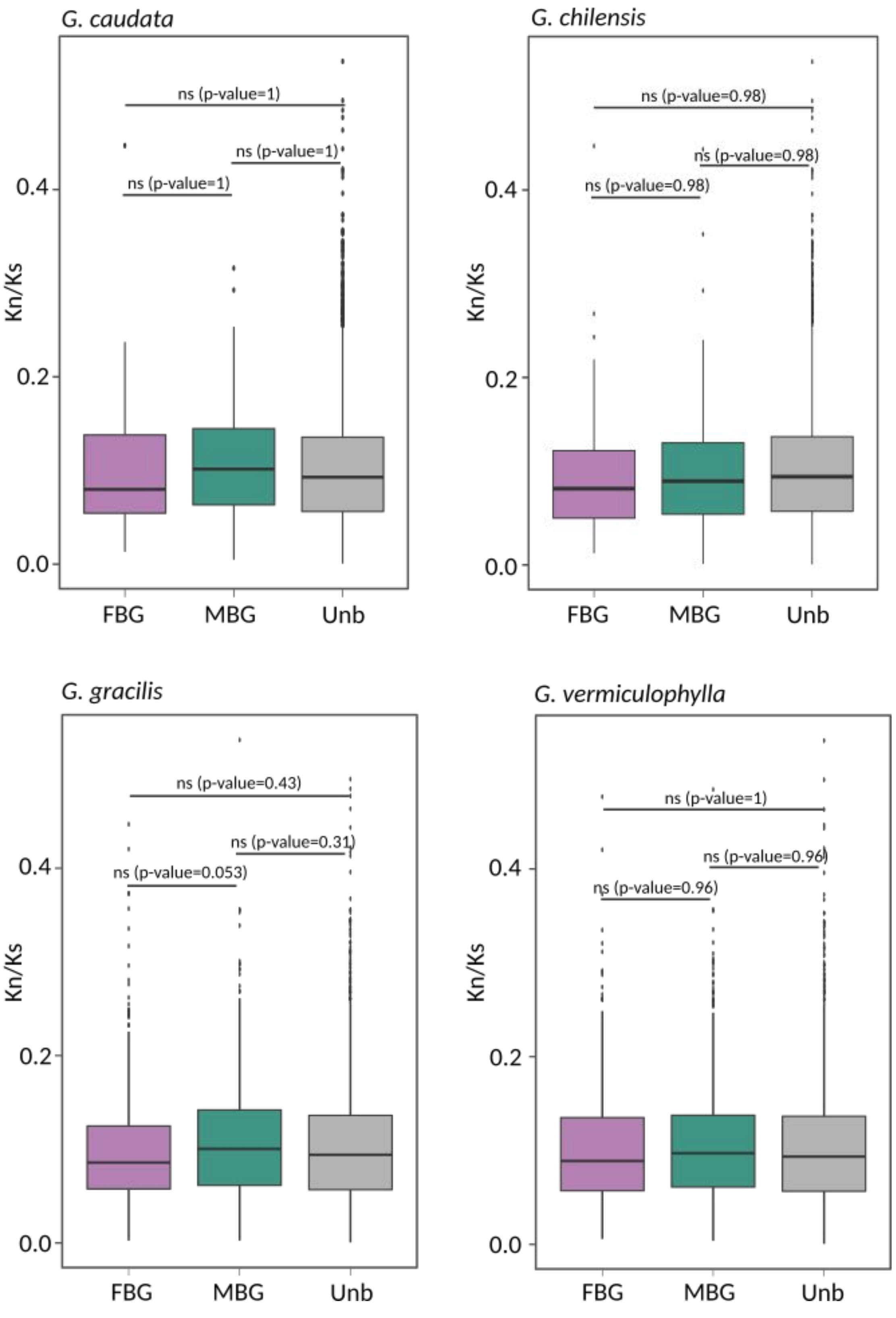

Fig. S6. Evolutionary rates (measured as K_N/K_S) of male- and female-biased genes compared to unbiased genes across all four *Gracilaria* species (codeml, model M0, PAML4) showing no statistical difference between the groups (pairwise Wilcoxon test with Holm correction).
